# Replacement of the Native Mitral Valve Due to Endocarditis Caused by *Stenotrophomonas Maltophilia*

**DOI:** 10.21470/1678-9741-2019-0300

**Published:** 2019

**Authors:** Ilker Alat, Ahmet Turhan Kılıç, Ersin Çelik

**Affiliations:** 1 Department of Cardiovascular Surgery, Afyonkarahisar State Hospital, Afyonkarahisar, Turkey.; 2 Department of Cardiovascular Surgery, Bursa State Hospital, Bursa, Turkey.; 3 Department of Cardiovascular Surgery, Isparta City Hospital, Isparta, Turkey.

**Keywords:** Endocarditis, Mitral Valve, Septic Shock, Stenotrophomonas maltophilia, Embolectomy

## Abstract

We report a case of a 59-year-old female patient with vegetative native mitral valve endocarditis caused by *Stenotrophomonas maltophilia* (SM). She had hemodialysis-dependent chronic renal failure, but no immunosuppressive disease. Echocardiography showed mobile vegetation on her native mitral valve. Right femoral artery embolectomy and mitral valve replacement were performed simultaneously. She awakened from anesthesia, but she passed away due to septic shock complications. To the best of our knowledge, this was the first case in whom native mitral valve endocarditis caused by SM was observed (despite of absence of any immunosuppressive event) and needed to undergo valve replacement.

**Table:** 

Abbreviations, acronyms & symbols	
CPB	= Cardiopulmonary bypass
SM	= *Stenotrophomonas maltophilia*
TMP-SMX	= Trimethoprim/sulfamethoxazole

## INTRODUCTION

*Stenotrophomonas maltophilia* (SM) is a gram-negative bacillus that is increasingly associated with serious nosocomial infections, especially in immunocompromised patients; however, the occurrence of endocarditis due to this organism is rare^[1]^. Reports of SM-caused endocarditis usually are related to either aortic valve prosthesis^[2]^ or mitral valve prosthesis^[3]^, or to patients who have a history of invasive procedures, such as pacemaker implantations^[4]^, or a history of accompanying immunosuppressive disease^[5]^. However, SM reporting as cause of endocarditis in the native mitral valve is extremely rare. Furthermore, in cases where SM endocarditis was reported in the native mitral valve, only medical treatment with antibiotics has been mentioned^[6]^. To the best of our knowledge, there is no other report in the literature in which it was required valve replacement surgery because of SM endocarditis in the patient’s native mitral valve although there was no immunosuppressive disease. It's herein presented a patient who underwent valve replacement and simultaneous femoral embolectomy operation due to SM endocarditis in her native mitral valve.

## CASE REPORT

**Fig. 1:**
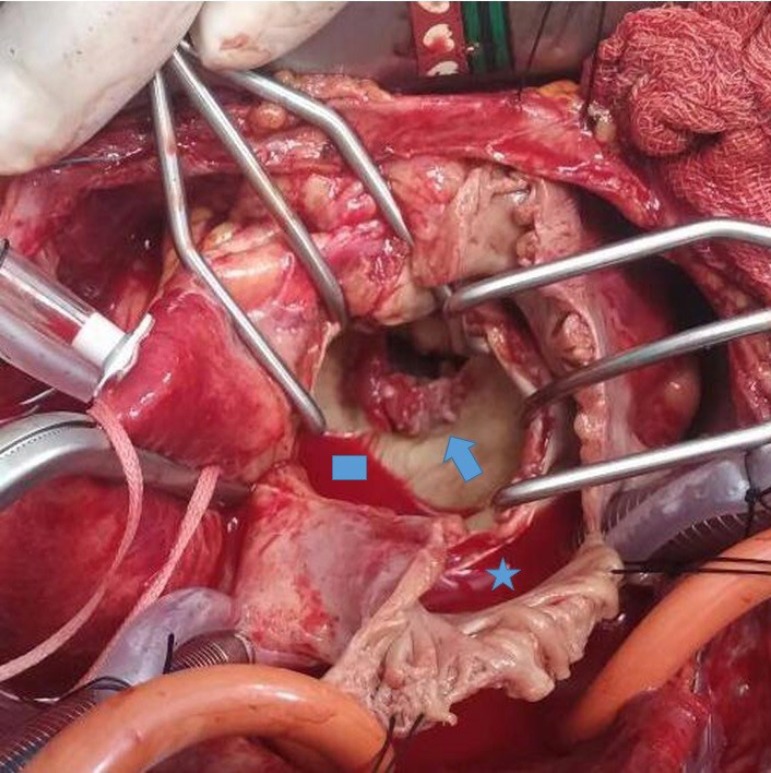
Intraoperative view of the native mitral valve with the vegetation on the posterior mitral leaflet. Left atrium and mitral valve are seen through the right atrial incision, consisting of a superior septal approach. The star indicates the right atrium, the rectangle indicates the left atrium, and the arrow indicates the vegetation on the posterior mitral leaflet caused by Stenotrophomonas maltophilia.

An ethical approval was obtained from the institutional board and authorities. A 59-year-old woman was admitted to the hospital with fever and blurred consciousness. The patient, with hemodialysis-dependent chronic renal failure for seven years, had been catheterized with a permanent hemodialysis catheter from the right internal jugular vein in an external center 10 days before. She was severely dyspneic and orthopneic. According to her relatives, she had fluctuations in her consciousness. She was sometimes able to recognize her relatives, and sometimes she could not do that. Additionally, her right lower extremity had been cold and cyanotic for three days. She had a loss of motion in her right lower extremity. Echocardiographic evaluation revealed mobile vegetation with an extremely fragile appearance on the posterior leaflet of mitral valve. The mitral valve opening was highly restricted due to vegetation on the leaflet. Her ejection fraction was 60% and pulmonary artery pressure was 50 mmHg. There was left atrial dilatation and a minimal tricuspid regurgitation. Despite of her dyspnea, her hemodynamic parameters allowed us to perform coronary angiography. There was no coronary lesion requiring surgery. The patient was operated as an emergency and right femoral embolectomy was performed simultaneously with mitral valve replacement. In regard to its macroscopic appearance, a similar material, like the one on the mitral valve, was taken out from the femoral artery. After the procedure, the blood flow in her lower extremity was normal. During the exploration, it was observed that the right atrial structures were clean and there was no vegetation on the tricuspid valve. Since everything had started with the last catheterization, we wanted to ensure all the right heart structures. Each fold, each structure, and all blind points at the first sight in the right heart (in both atrium and ventricle) were examined carefully. Interestingly, nothing wrong was observed. Similar examinations were carried out for the left heart. This was important because, she had a femoral embolus and possibly cerebrovascular events due to this mobile vegetation. So, through the incisions and resected mitral valve area, the structures of the whole left heart were examined carefully. The difference of this operation from a classical endocarditis surgery was that the patient had a history of embolism in two separate systems (leg and brain). This forced the surgical team to make sure that all the chambers/folds in the heart were clean. Cross-clamp and cardiopulmonary bypass (CPB) times were 160 mins and 182 mins, respectively. She did not need any kind of circulatory assistance equipment during or after the surgery. However, the patient was under inotropic drugs support during the surgery after CPB and in her postoperative period. As it can be seen in Figure 1, when mitral valve was explored through superior septal approach, fragile vegetation was observed on posterior mitral leaflet. After resection of the native valve, mechanical mitral valve replacement was performed with a #27 Sorin-Carbomedics mitral valve. Both the vegetation materials on leaflet and the femoral embolectomy materials were sent for microbiological examination. The patient was taken to the intensive care unit after the operation and hemodialysis was performed. Since the preoperative period, an empirical antibiotic treatment was started by infectious diseases specialists. The patient consciously awakened in the intensive care unit. As a result of microbiological studies, which were performed on hemoculture samples obtained during her admittance in the infectious disease clinic three days before, SM was isolated and it was resistant to all antibiotics, except for trimethoprim/sulfamethoxazole (TMP-SMX). The antibiotic regimen was converted to TMP-SMX, but due to septic shock complications, like severe hypotension unresponsive to continuous positive inotropic and vasoconstrictor intravenous drug therapy, she passed away on the postoperative first day.

## DISCUSSION

SM is a commensal and an emerging pathogen earlier noted in broad-spectrum life-threatening infections among vulnerable people, but more recently as a pathogen in immunocompetent individuals. According to previous reports, SM can lead to necrotizing otitis, cutaneous infections, endocarditis, meningitis, acute respiratory tract infections, bacteremia, tropical pyomyositis, cystic fibrosis, and septic arthritis^[7]^. However, when the literature is examined carefully, it is observed that endocarditis caused by this bacterium is extremely rare^[1]^ and especially includes a certain group of patients. This particular group of patients includes those with aortic or mitral prosthetic valve replacement and those with immune deficiency for any reason^[2,3,5]^. SM-caused endocarditis of native mitral valve is quite rare in the literature. Moreover, when the reports of these cases are examined, it is seen that they were subjected to a previous treatment, such as balloon mitral valvuloplasty, even if there is a native mitral valve^[6]^. If we specify precisely, to the best of our knowledge, the patient in this article is the first report in the literature regarding to three different aspects:

1 - This is the first patient in the literature who underwent mitral valve replacement surgery due to SM endocarditis although he/she had previously untouched native mitral valve.
2 - This is the first patient in the literature who underwent femoral embolectomy operation due to septic embolism caused by SM. Additionally, fluctuations of her consciousness could be a result of septic cerebrovascular embolism.
3 - Figure 1 is the first intraoperative photograph in the literature exhibiting the vegetation caused by SM.

Due to these three features mentioned above, this case is believed to have an important place in the literature.

As mentioned in the previous literature^[7]^, SM is truly a life-threatening microorganism. Unfortunately, using even today's state-of-the-art technologies, like open-heart surgeries, to treat SM-induced endocarditis is not enough to be successful. There are two main reasons for this failure:

α - As seen in most of the previous reports in the literature, SM is often and only sensitive to TMP-SMX. According to Shah et al.^[8]^, minocycline can be an alternative choice for the treatment of SM endocarditis. However, TMP-SMX was the only effective antibiotic in our culture antibiogram. There was no minocycline among the 19 antibiotics in our culture antibiogram. As a result, SM is resistant to a large number of other antibiotics present in a conventional antibiogram report. However, TMP-SMX is not the first choice of conventional empirical treatment of endocarditis. And the rarity of the incidence does not justify the idea that TMP-SMX should be an additional agent in each case of endocarditis. Facing a case of endocarditis, a physician prefers to proceed with antibiotics that are believed to be much stronger. This contradiction makes the body vulnerable to SM and the microorganism finds easier to operate.
β - A further disadvantage is that the time taken for the culture and culture antibiogram is long enough to facilitate the ability of SM to form the septic events. However, for the time being, it does not look like it is possible to avoid. With the studies to be carried out in this regard, early detection may be possible in the future with the development of tests that perform several antigen measurements that may be specific for SM. In this way, waiting for hours instead of days for the result can really be lifesaving.

SM endocarditis is a serious condition that is progressively rapid and fatal despite the surgical treatment choices, like open-heart surgeries. The only satisfactory thing about endocarditis caused by this microorganism is that it is rarely seen. It is important to work on rapid diagnostic methods that will be developed specifically for this microorganism. It's believed that this article will encourage researchers in this direction.

**Table:** 

Authors' roles & responsibilities	
IA	Substantial contributions to the conception or design of the work; or the acquisition, analysis, or interpretation of data for the work; final approval of the version to be published
ATK	Substantial contributions to the conception or design of the work; or the acquisition, analysis, or interpretation of data for the work; final approval of the version to be published
EÇ	Substantial contributions to the conception or design of the work; or the acquisition, analysis, or interpretation of data for the work; final approval of the version to be published
